# A seed sequence variant in miR-145-5p causes multisystem smooth muscle dysfunction syndrome

**DOI:** 10.1172/JCI166497

**Published:** 2023-03-01

**Authors:** Christian Lacks Lino Cardenas, Lauren C. Briere, David A. Sweetser, Mark E. Lindsay, Patricia L. Musolino

**Affiliations:** 1 Cardiovascular Research Center, Massachusetts General Hospital, Boston, Massachusetts, USA.; 2 Harvard Medical School, Boston, Massachusetts, USA.; 3 Division of Genetics,; 4 Undiagnosed Disease Network,; 5 Center for Genomic Medicine,; 6 Cardiovascular Genetics Program, and; 7 Department of Neurology, Massachusetts General Hospital, Boston, Massachusetts, USA.

**Keywords:** Vascular Biology, Cardiovascular disease, Stroke

**To the Editor:** Multisystemic smooth muscle dysfunction syndrome (MSMDS, OMIM #613834) is an ultrarare smooth muscle myopathy (1). Cases are monogenic from missense variation at arginine 179 of the *ACTA2* gene (1, 2). Herein, we describe a case of MSMDS associated with a single-nucleotide variant in the gene *MIR145*.

Fetal ultrasound revealed polyhydramnios, enlarged abdomen and bladder, and prune belly syndrome. Profound gastrointestinal dysmotility was identified during infancy. His cerebrovascular disease began with frontal cortex and watershed strokes at approximately 2.5 years of age. Straightening of cerebral arteries and flattening of the genu of the corpus callosum and pons was observed. During school age he had multiple strokes consistent with arterial ischemic and watershed infarctions. Severe progressive steno-occlusive disease developed, which was worse in the anterior circulation (Figure 1, A and B). The vascular anatomy also showed straightening and decreased caliber of the terminal internal carotid artery, consistent with described cases of MSMDS (3, 4). Thoracic aortic imaging has been normal.

A thoracic aortic aneurysm/dissection panel was negative, including analysis of *ACTA2*. Quad genome sequencing was negative; however, research-based analysis revealed a de novo single-nucleotide variant in *MIR145* (NR_029686.1: n.18C>A) (Figure 1C). This variant is absent from gnomAD, has a CADD score of 20.9, and *MIR145* is enriched in tissues with high smooth muscle cell (SMC) content (5) (Supplemental Figure 1; supplemental material available online with this article; https://doi.org/10.1172/JCI166497DS1). The *MIR145* transcript is processed into 2 microRNAs (miRs), with the variant position at nucleotide 3 of miR-145-5p.

To determine whether the miR-145-5p variant could mediate the observed patient phenotype of smooth muscle dysfunction, we undertook molecular analysis. Cases of MSMDS to date have been caused by recurrent missense variants in the *ACTA2* gene, altering arginine 179 (2). These variants impair α-smooth muscle actin (α-SMA) function, resulting in a cellular state resembling a loss of protein function. The miR-145-5p variant is located within the seed sequence (nt 2–8), the portion of a miR that stalls lateral diffusion of the RISC complex and promotes stable interactions with complementary RNAs (Figure 1C). We hypothesized that mutant miR-145-5p may not be able to target 3′ UTRs that mediate proper SMC function and may thus result in cellular changes similar to *ACTA2* R179 variants. To assess this possibility, we exposed human vascular SMCs to an siRNA targeting miR-145-5p, wild-type (WT) miR-145-5p, or a mutant version of miR-145-5p with the patient variant. Indeed, transfection of either an siRNA against miR-145-5p or mutant miR-145-5p induced a notable decrease in the expression of several cytoskeletal proteins, including transgelin, calponin, and importantly, α-SMA (Figure 1D and Supplemental Figure 2; see complete unedited blots in the supplemental material).

Cellular models of the *ACTA2* R179H mutation demonstrate global filamentous actin (F-actin) cytoskeletal deficiency (6). Transfection of either siRNA against miR-145-5p or the mutant miR-145-5p induced a phenotype characterized by deficient F-actin, whereas treatment with WT miR-145-5p enhanced stress fiber formation (Figure 1E). Therefore, we next performed RNA-seq analysis that included mRNAs and miRs in patient skin fibroblasts and compared them to WT skin fibroblasts. Principal component analysis of differentially expressed genes (DEGs) substantially differentiated the patient’s fibroblasts from control fibroblasts (Supplemental Figure 2). Furthermore, pathway analysis of DEGs showed enrichment of categories related to “hsa04810: regulation of actin cytoskeleton” (Supplemental Figure 3). Hybridization analysis and miR RNA-seq demonstrated a decrease in expression of miR-145-5p in the presence of mutant miR-145-5p (Supplemental Figures 1 and 4), consistent with impairment in a positive feedback loop for *MIR145* expression (5).

In conclusion, genetic variation in the *MIR145* gene expands the possible loci associated with MSMDS and further confirms the syndrome as a disorder of failed SMC development and function, although discovery of further cases will be necessary to confirm our findings. To our knowledge this is the first patient reported with a monogenic vascular disease caused by a mutation in a noncoding gene.

## Supplementary Material

Supplemental data

## Figures and Tables

**Figure 1:**
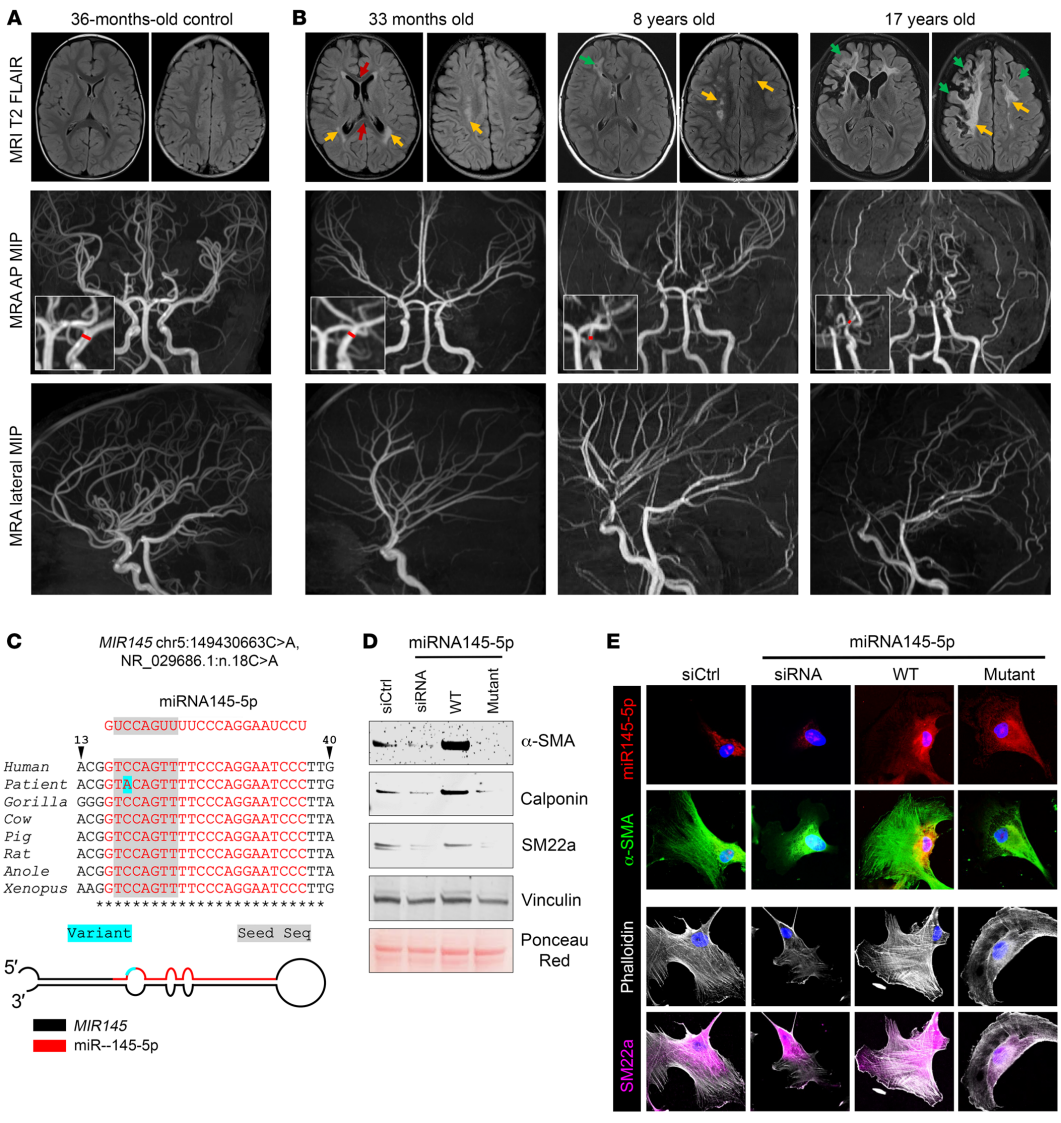
Representative MRI T2-weighted FLAIR and maximum intensity projection (MIP) of MR angiography (MRA) images. (**A**) Images from a 36-month-old control and (**B**) 3 different time points of patient with miR-145-5p mutation. MRA images are anterior-posterior [AP] and lateral MIP. Note increased angulation of the forceps of the corpus callosum (red arrows) and significant bilateral periventricular and right watershed white matter injury (yellow arrows) at age 33 months, which progressed to bilateral white matter (yellow arrows) and right frontal ischemic infarctions (green arrows) by 8 years of age. Additional arterial ischemic infarctions occurred through 17 years of age. Vascular anatomy showed straightening and decreased caliber of the terminal internal carotid artery (ICA) and basal cerebral arteries with progression of the relative stenosis of the terminal ICA (red bar) when compared with its petrous segment. (**C**) Variant in the *MIR145* gene shown as primary structure in multiple species comparison and in secondary structure. (**D** and **E**) Vascular SMCs transduced with indicated miRs, analyzed by Western blotting and immunofluorescence, demonstrate that the mutant version of miR-145-5p fails to mediate contractile protein expression or induce stress fiber formation similar to WT miR-145-5p. *Sequence conservation across species.

## Version 1 01/17/2023

In-Press Preview

## Version 2 03/01/2023

Electronic publication
