# Supplementary material for: A seed sequence variant in miR-145-5p causes multisystem smooth muscle dysfunction syndrome
Source: J Clin Invest. 2023 Mar 1;133(5):e166497. doi: 10.1172/JCI166497 (PMC9974090; doi:10.1172/JCI166497)

Supplemental Data

A Seed Sequence Variant in miR-145-5p causes Multisystem Smooth Muscle Dysfunction Syndrome (MSMDS)

Christian Lacks Lino Cardenas[1,7,ϯ], Lauren C. Briere[3,4,6,ϯ], Undiagnosed Diseases Network[§], David A. Sweetser[3,4,7], Mark E. Lindsay[1,2,7], Patricia L. Musolino[5,6,7]

[1]Cardiovascular Research Center and [2]Cardiovascular Genetics Program,
Division of Cardiology, Department of Medicine, Massachusetts General Hospital
[3]Division of Genetics, Department of Pediatrics, Massachusetts General Hospital
[4]Undiagnosed Disease Network, Massachusetts General Hospital
[5]Department of Neurology, Massachusetts General Hospital
[6]Center for Genomic Medicine, Massachusetts General Hospital
[7]Harvard Medical School

[ϯ]These authors contributed equally

Contents:

Supplemental Figures 1-4

Methods

[§]List of Members of the Undiagnosed Diseases Network

Supplemental Figure 1

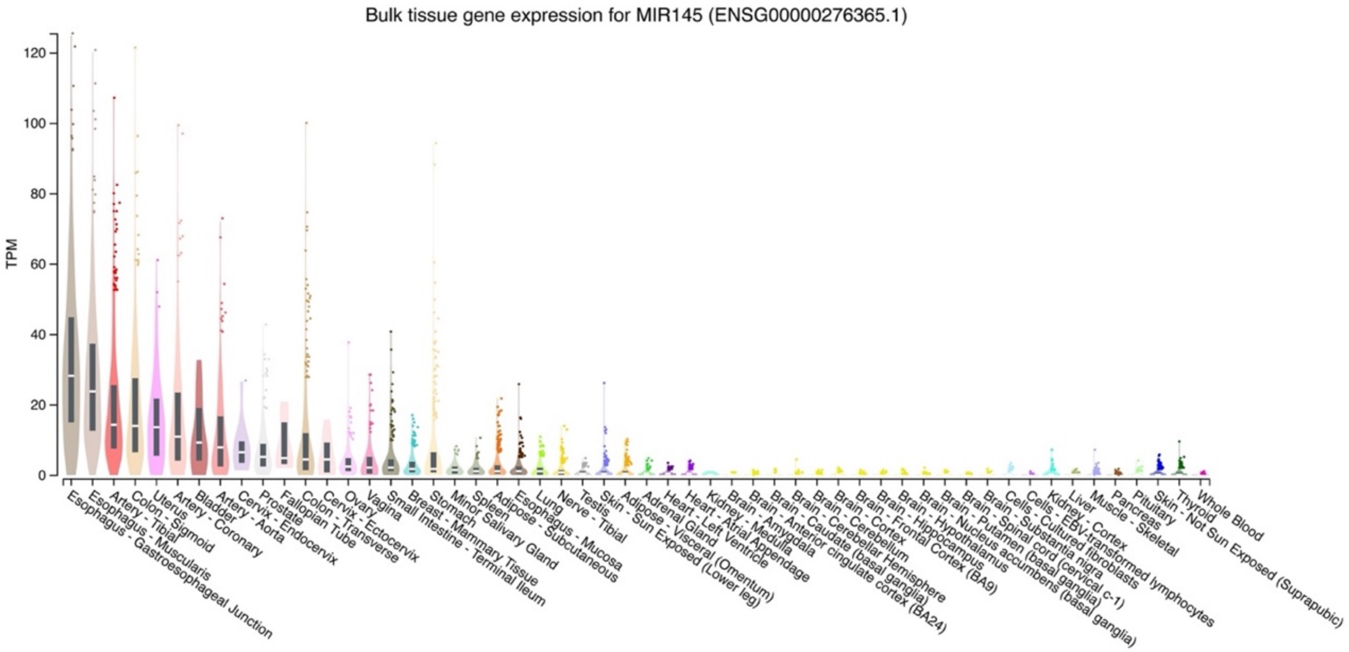

:.

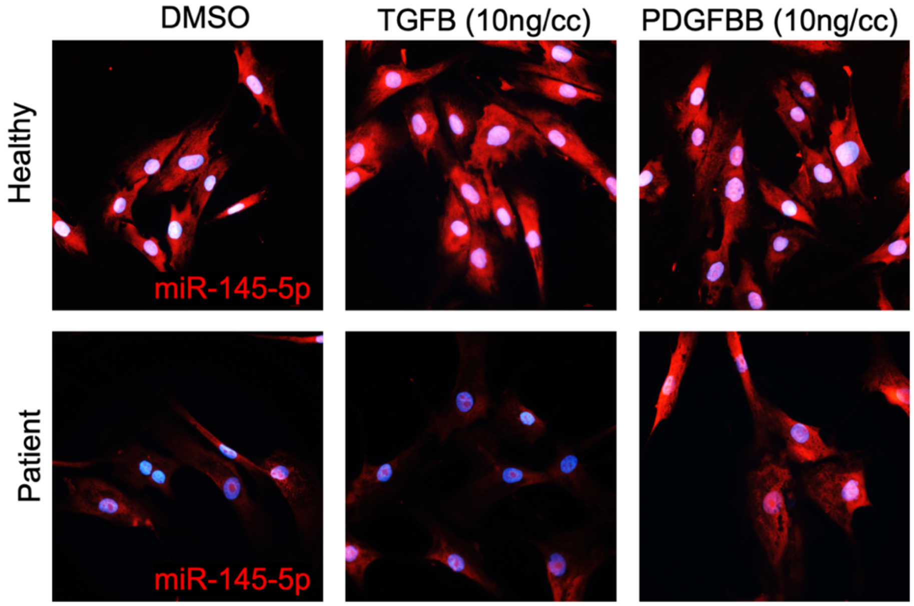

Supplemental Figure 1: (Top) Bulk tissue RNA analyzed for *MIR145* demonstrates enrichment in organs with high smooth muscle enrichment including esophagus, colon, uterus, bladder and arterial tissues. This figure was obtained from the GTEx Portal on 09/01/2022. (Bottom) *In Situ* hybridization of mIR-145-5p in control and patient skin fibroblasts. Fibroblasts were exposed to 5ng/cc of either transforming growth factor beta-1 (TGFB1) or platelet derived growth factor BB (PDGFBB) for 12 hours prior to fixation and staining as indicated.

Supplemental Figure 2

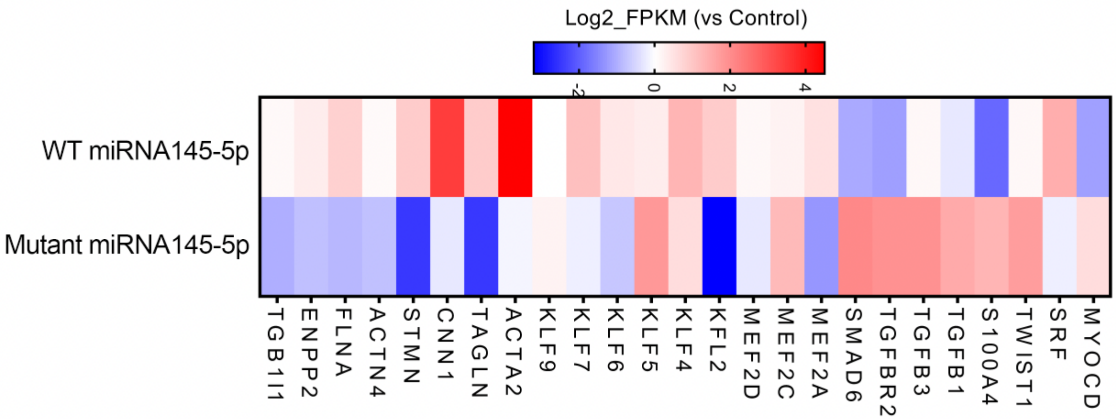

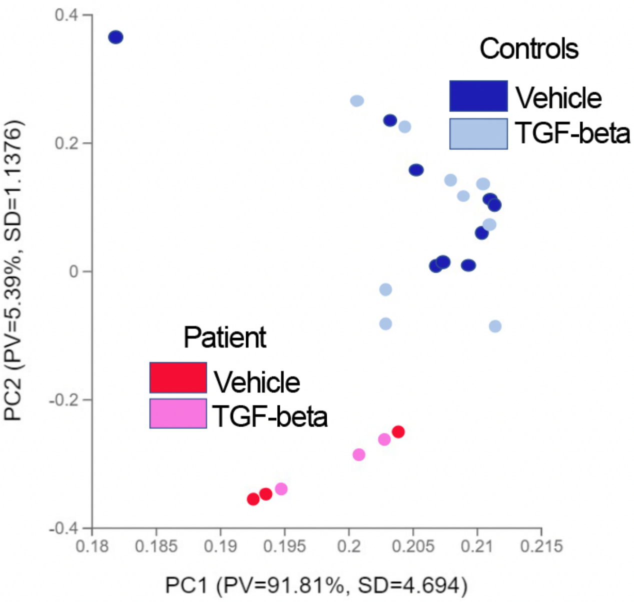

Supplemental Figure 2: (Top) Heat diagram of selected vascular transcripts from human vascular smooth muscle cells exposed to either wild type or mutant miRNA145-5p both compared VSMCs treated with scramble mIR (labeled as Control). (Bottom) PCA analysis of fibroblasts from the patient and several control fibroblast lines show differentiation of the patient’s transcriptome from normal fibroblasts.

Supplemental Figure 3

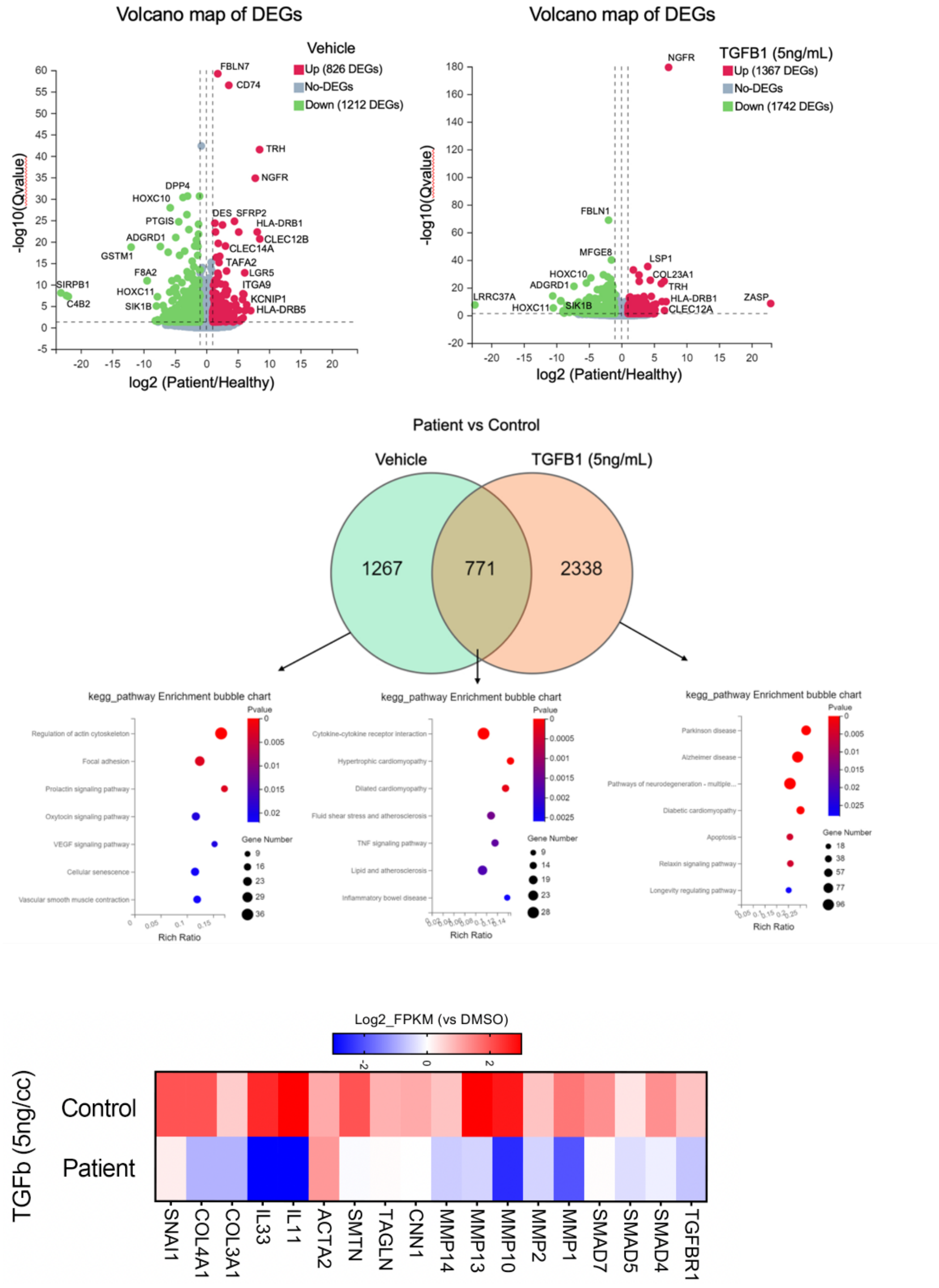

Supplemental Figure 3: (Top) Volcano plot highlighting the top differentially expressed genes in patient fibroblasts compared to control fibroblasts either unstimulated or exposed to 5ng/cc TGFB1 for 12 hours prior to harvest. (Middle) Venn diagram and Kegg pathway analysis of significant DEGs according to TGFB stimulation status. (Bottom) Heat diagram of selected vascular genes in patient fibroblasts and control fibroblasts in cell exposed to 5ng/cc TGFB1 compared to DMSO exposure.

Supplemental Figure 4

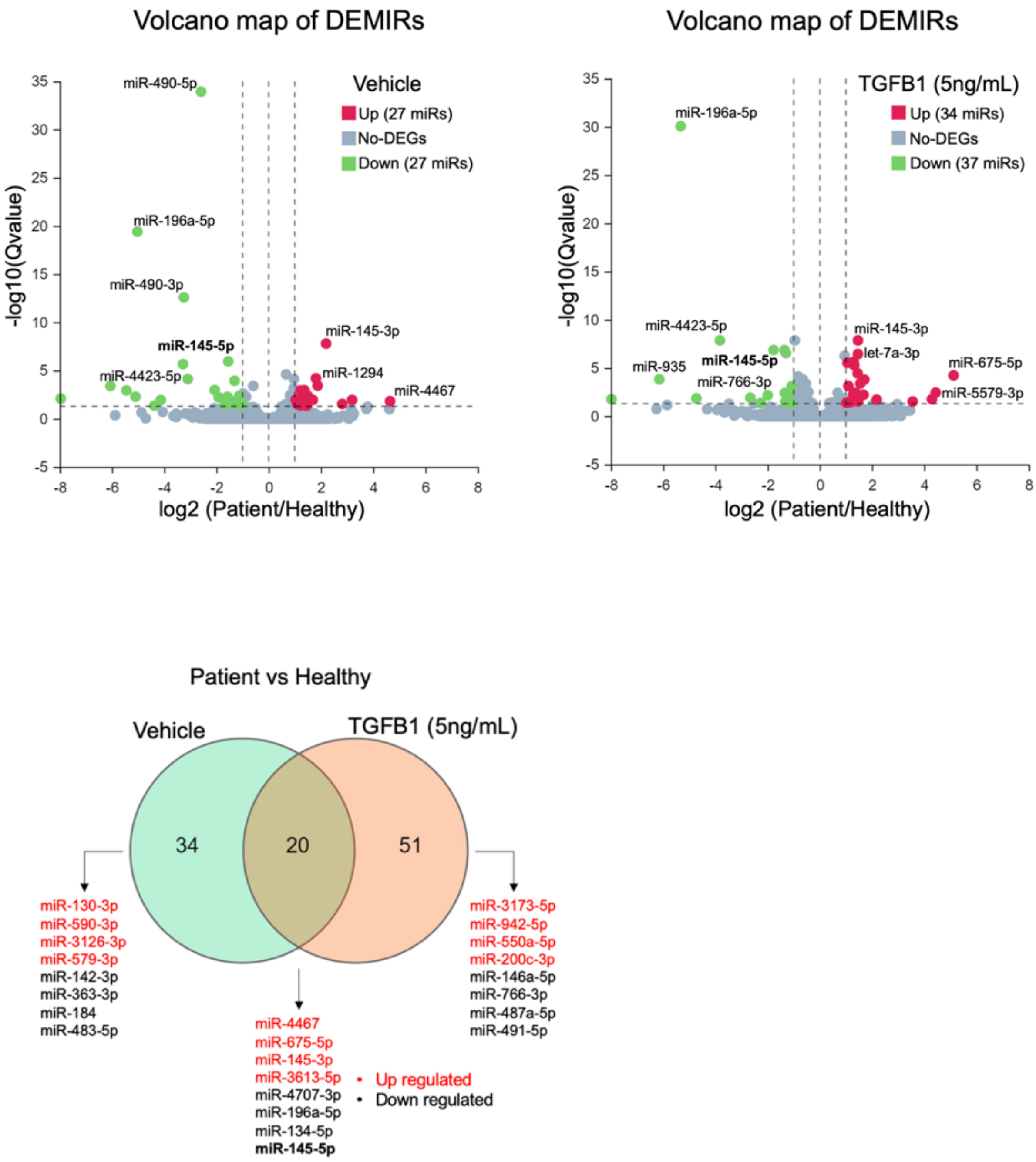

Supplemental Figure 4: (Top) Volcano plot highlighting the top differentially expressed miRNAs in patient fibroblasts compared to control fibroblasts either unstimulated or exposed to 5ng/cc TGFB1 for 12 hours prior to harvest. miR-145-5p is significantly downregulated in the patient in both conditions (bold). (Bottom) Venn diagram of significant DEGs according to TGFB stimulation status.

Methods:

Human Subjects: Written informed consent was obtained from the participant's guardians. The participant and his family were enrolled in the Undiagnosed Diseases Network (UDN) study, which was approved by the National Institutes of Health Intramural Institutional Review Board. The participant was also enrolled in the study The Young Genetic Stroke Alliance: a Natural History with Biomarkers of Hereditary Cerebrovascular Disorders, which was approved by the Mass General Brigham Institutional Review Board.

Cell lines. Primary human aortic vascular smooth muscle cells (VSMC) were purchased from Cell Applications Inc. (354K-05a), California, USA. To preserve cell identity all experiments were carried out at passages 1-5, and cell identity (contractile phenotype) was assessed by immunofluorescence staining of contractile markers including SM22α, α-SMA, MYH11 and cytoskeleton integrity by F-actin staining. Human fibroblasts from healthy and patient individuals were obtained. Tissue biopsies were dissected into small blocks and placed in appropriate culture dishes and supplemented with growth media. Tissue was cultured for 2-3 weeks until a single cell monolayer covered the surface of the dish followed by passage of cells to perform experiments.

miRNA overexpression. Both wildtype (GUCCAGUUUUCCCAGGAAUCCU) and mutant (GUACAGUUUUCCCAGGAAUCCU) Hsa-miR145-5p mimics were synthesized by thermo Fisher Scientific Asheville LLC. Then 40nM of each miR145-5p were transfected into human primary vascular smooth muscle cells using Lipofectamine™ RNAiMAX Transfection (Cat. #13778030) and Opti-MEM reduced serum medium at 5µl/ml (Cat. #31985070) for 16 hrs. After 48 hrs post transfection, cells were prepared for either total RNA or protein lysate extraction as indicated below.

miR145-5p inhibition. Healthy VSMCs were transfected with 30 nM of siCtrl (Thermo Fisher Scientific, Cat. #AM17010) or anti-miR145-5p (Thermo Fisher Scientific, Cat. #AM14000) for 48 hours using Lipofectamine™ RNAiMAX Transfection (Thermo Fisher Scientific, Cat. #13778030) at 5µl/ml, followed by 24 hours of normal growing medium. Then, 30 µg of total protein was prepared from siRNA-treated cells nucleic acid and immunoblot analysis.

RNA isolation and RNA-seq analysis. Total large and small RNAs were extracted using a RNeasy kit (Qiagen, Cat. No. / ID: 217084) and miReasy Advanced kit (Qiagen, Cat. No. / ID: 217684) following the manufacturer's protocol. For RNA-seq of both skin fibroblasts and VSMCs (human primary smooth muscle cells), we used the BGISEQ platform, on average generating about 4.55G (human) Gb bases per sample. The average alignment ratio with the reference genome was 97.99% for humans. A total of 17582 genes were identified. For microRNA-seq A total of 24 samples were sequenced on DNBSEQ platform, with an average yield of 26.57 M reads per sample. The average alignment ratio of the sample comparison genome was 94.64%. A total of 1931 miRNAs were detected. We used HISAT to align the clean reads to the reference genome and Bowtie2 to align the clean reads to the reference genes. In order to reflect the correlation of gene expression between samples, the Pearson correlation coefficients of all gene expressions between each two samples were calculated, and these coefficients were reflected in the form of a heatmap. The correlation coefficients can reflect the similar situation of the overall gene expression between each sample. The higher the correlation coefficient is, the more similar the gene expression level was. The raw dataset is available at the National Center for Biotechnology Information's Gene Expression Omnibus Database (accession no. GSE220038).

Immunoblotting. Protein lysates were extracted using RIPA buffer (ThermoFisher, CA, USA) and supplemented with 1× of protease inhibitor cocktail (Roche) according to the manufacturer's instruction. 20 µg of total extracts were mixed with a denaturing buffer (1× Laemmli loading buffer with 10% of β-mercaptoethanol) and analyzed by SDS–PAGE/western blot. Separated proteins were transferred onto a nitrocellulose membrane using the iBlot transfer system (Novex,ThermoFisher, USA). In general, primary antibodies were used at concentration of 1:100 and secondary at concentration of 1:10000. α-Smooth Muscle Actin (Cell Signaling, (D4K9N) XP® Rabbit mAb

#19245), Calponin 1 (Abcam, rabbit monoclonal, [EP798Y]-ab46794), TAGLN/Transgelin (Abcam, rabbit polyclonal, ab14106), Vinculin (Santa Cruz biotechnologies (7F9)-sc-73614).

In situ hybridization and immunofluorescence. Qiagen FISH miR-145-5p Probe labeled with Quasar® 570-labeled oligos (Biosearch Technologies, Inc.Petaluma, CA) were hybridized to VSMCs followed by incubation following the manufacturer's instructions. For immunofluorescence staining of cytoskeletal markers. For immunocytochemistry, cells were cultured into 8-well Lab-TekTM II Chamber Slides (NuncTM). Cells were rinsed twice with ice-cold PBS, fixed with 4% paraformaldehyde in PBS (PFA, Boston Bioproducts) for 15 min at rt, and were permeabilized with 0.1% Triton-X (Sigma–Aldrich) for 10 min. The slides were blocked with 10% donkey-serum in PBS-Tween 20 (0.1%) for 1 h at rt. Subsequently, the antibodies anti-SM22a (1:100), anti-alpha-SMA (1:50) and anti-F-actin (1:50) were added and slides were incubated overnight at 4°C. The slides were then washed 3 times for 5 min each with PBS-T and were incubated with secondary antibody. for 1 hour at room tempature. Slides were visualized with the Leica TCS SP8 confocal microscopy station and micrographs were digitized with the Leica Application Suite X software.

Members of the Undiagnosed Diseases Network

Maria T. Acosta
Margaret Adam
David R. Adams
Justin Alvey
Laura Amendola
Ashley Andrews
Euan A. Ashley
Mahshid S. Azamian
Carlos A. Bacino
Guney Bademci
Ashok Balasubramanyam
Dustin Baldridge
Jim Bale
Michael Bamshad
Deborah Barbouth
Pinar Bayrak-Toydemir
Anita Beck
Alan H. Beggs
Edward Behrens
Gill Bejerano
Hugo J. Bellen
Jimmy Bennet
Beverly Berg-Rood
Jonathan A. Bernstein
Gerard T. Berry
Anna Bican
Stephanie Bivona
Elizabeth Blue
John Bohnsack
Devon Bonner
Lorenzo Botto
Brenna Boyd
Lauren C. Briere
Elly Brokamp
Gabrielle Brown
Elizabeth A. Burke
Lindsay C. Burrage
Manish J. Butte
Peter Byers
William E. Byrd
John Carey
Olveen Carrasquillo
Thomas Cassini
Ta Chen Peter Chang
Sirisak Chanprasert
Hsiao-Tuan Chao
Gary D. Clark
Terra R. Coakley
Laurel A. Cobban
Joy D. Cogan
Matthew Coggins
F. Sessions Cole
Heather A. Colley
Cynthia M. Cooper

Heidi Cope
William J. Craigen
Andrew B. Crouse
Michael Cunningham
Precilla D'Souza
Hongzheng Dai
Surendra Dasari
Joie Davis
Jyoti G. Dayal
Matthew Deardorff
Esteban C. Dell'Angelica
Katrina Dipple
Daniel Doherty
Naghmeh Dorrani
Argenia L. Doss
Emilie D. Douine
Laura Duncan
Dawn Earl
David J. Eckstein
Lisa T. Emrick
Christine M. Eng
Cecilia Esteves
Marni Falk
Liliana Fernandez
Elizabeth L. Fieg
Paul G. Fisher
Brent L. Fogel
Irman Forghani
William A. Gahl
Ian Glass
Bernadette Gochuico
Rena A. Godfrey
Katie Golden-Grant
Madison P. Goldrich
Alana Grajewski
Irma Gutierrez
Don Hadley
Sihoun Hahn
Rizwan Hamid
Kelly Hassey
Nichole Hayes
Frances High
Anne Hing
Fuki M. Hisama
Ingrid A. Holm
Jason Hom
Martha Horike-Pyne
Alden Huang
Yong Huang
Wendy Introne
Rosario Isasi
Kosuke Izumi
Fariha Jamal
Gail P. Jarvik
Jeffrey Jarvik
Suman Jayadev

Orpa Jean-Marie
Vaidehi Jobanputra
Lefkothea Karaviti
Jennifer Kennedy
Shamika Ketkar
Dana Kiley
Gonench Kilich
Shilpa N. Kobren
Isaac S. Kohane
Jennefer N. Kohler
Deborah Krakow
Donna M. Krasnewich
Elijah Kravets
Susan Korrick
Mary Koziura
Seema R. Lalani
Byron Lam
Christina Lam
Grace L. LaMoure
Brendan C. Lanpher
Ian R. Lanza
Kimberly LeBlanc
Brendan H. Lee
Roy Levitt
Richard A. Lewis
Pengfei Liu
Xue Zhong Liu
Nicola Longo
Sandra K. Loo
Joseph Loscalzo
Richard L. Maas
Ellen F. Macnamara
Calum A. MacRae
Valerie V. Maduro
Rachel Mahoney
Bryan C. Mak
May Christine V. Malicdan
Laura A. Mamounas
Teri A. Manolio
Rong Mao
Kenneth Maravilla
Ronit Marom
Gabor Marth
Beth A. Martin
Martin G. Martin
Julian A. Martínez-Agosto
Shruti Marwaha
Jacob McCauley
Allyn McConkie-Rosell
Alexa T. McCray
Elisabeth McGee
Heather Mefford
J. Lawrence Merritt
Matthew Might
Ghayda Mirzaa
Eva Morava

Paolo M. Moretti
Mariko Nakano-Okuno
Stan F. Nelson
John H. Newman
Sarah K. Nicholas
Deborah Nickerson
Shirley Nieves-Rodriguez
Donna Novacic
Devin Oglesbee
James P. Orengo
Laura Pace
Stephen Pak
J. Carl Pallais
Christina GS. Palmer
Jeanette C. Papp
Neil H. Parker
John A. Phillips III
Jennifer E. Posey
Lorraine Potocki
Barbara N. Pusey
Aaron Quinlan
Wendy Raskind
Archana N. Raja
Deepak A. Rao
Anna Raper
Genecee Renteria
Chloe M. Reuter
Lynette Rives
Amy K. Robertson
Lance H. Rodan
Jill A. Rosenfeld
Natalie Rosenwasser
Francis Rossignol
Maura Ruzhnikov
Ralph Sacco
Jacinda B. Sampson
Mario Saporta
Judy Schaechter
Timothy Schedl
Kelly Schoch
C. Ron Scott
Daryl A. Scott
Vandana Shashi
Jimann Shin
Edwin K. Silverman
Janet S. Sinsheimer
Kathy Sisco
Edward C. Smith
Kevin S. Smith
Emily Solem
Lilianna Solnica-Krezel
Ben Solomon
Rebecca C. Spillmann
Joan M. Stoler
Jennifer A. Sullivan
Kathleen Sullivan

Angela Sun
Shirley Sutton
David A. Sweetser
Virginia Sybert
Holly K. Tabor
Amelia L. M. Tan
Queenie K.-G. Tan
Mustafa Tekin
Fred Telischi
Willa Thorson
Cynthia J. Tifft
Camilo Toro
Alyssa A. Tran
Brianna M. Tucker
Tiina K. Urv
Adeline Vanderver
Matt Velinder
Dave Viskochil
Tiphanie P. Vogel
Colleen E. Wahl
Melissa Walker
Stephanie Wallace
Nicole M. Walley
Jennifer Wambach
Jijun Wan
Lee-kai Wang
Michael F. Wangler
Patricia A. Ward
Daniel Wegner
Monika Weisz-Hubshman
Mark Wener
Tara Wenger
Katherine Wesseling Perry
Monte Westerfield
Matthew T. Wheeler
Jordan Whitlock
Lynne A. Wolfe
Kim Worley
Changrui Xiao
Shinya Yamamoto
John Yang
Diane B. Zastrow
Zhe Zhang
Chunli Zhao
Stephan Zuchner

Lino Cardenas et al., A Seed Sequence Variant in miR-145-5p causes Multisystem Smooth Muscle Dysfunction Syndrome (MSMDS)

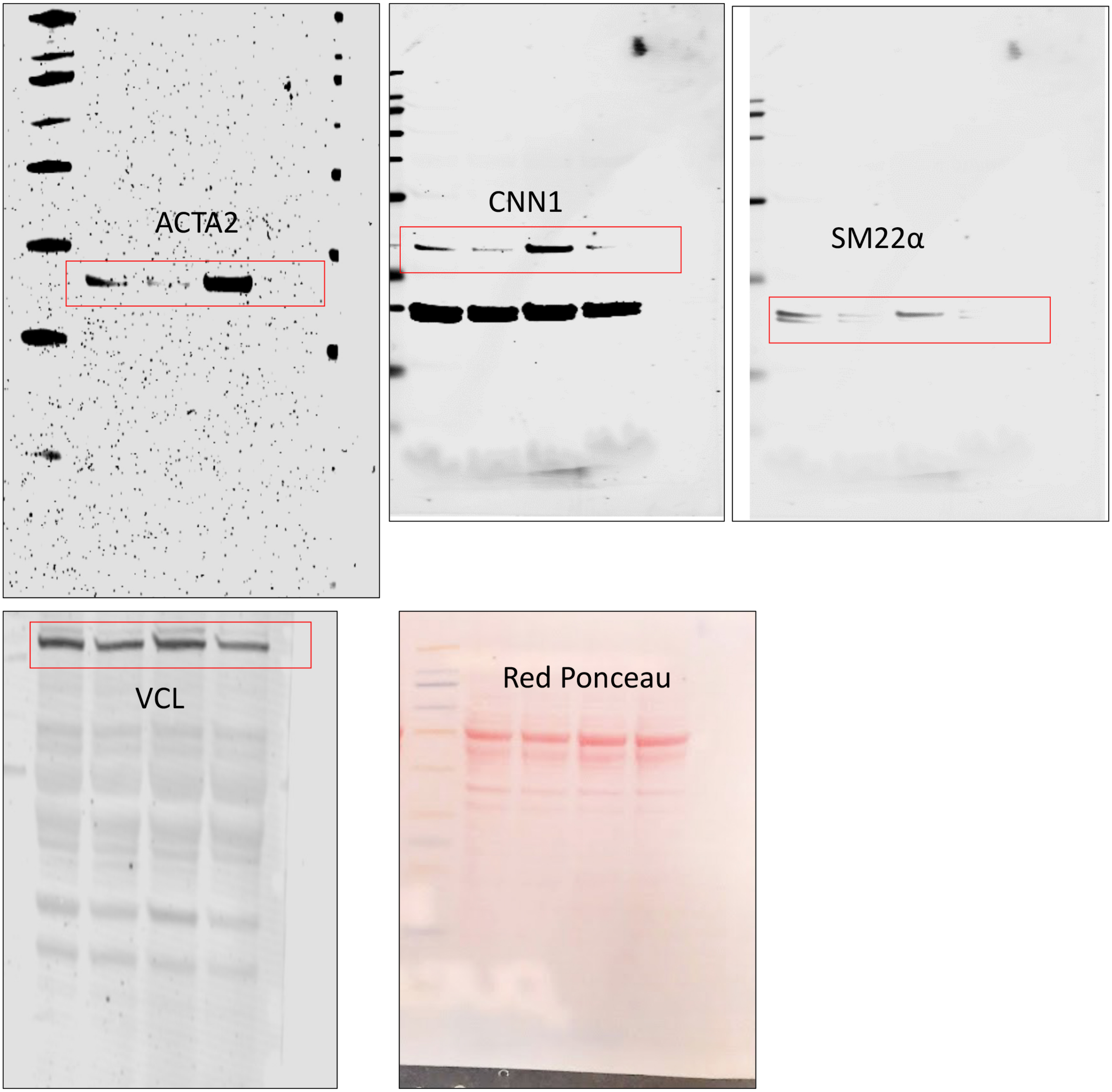

Supplement: Supplemental data [file jci-133-166497-s086.pdf]
